# Nexus among indigenous languages, agricultural radio programmes and behavioural change towards agricultural practices in Nigeria

**DOI:** 10.1016/j.heliyon.2024.e33721

**Published:** 2024-06-29

**Authors:** Babatunde Adeyeye, Abiodun Salawu, Evaristus Adesina

**Affiliations:** Indigenous Language Media in Africa Research Entity, Northwest University, Mafikeng Campus, South Africa

**Keywords:** Agriculture, Behavioural change, Communication, Indigenous languages, Radio

## Abstract

This study investigated the interrelationship between indigenous languages, agricultural radio programmes and behavioural change towards agricultural practices in Nigeria, focusing on three states (Benue, Nasarawa and Plateau). The following research objectives guided the study: to ascertain farmers' awareness of radio programmes in indigenous languages, identify farmers' sources of information, ascertain farmers' access to agricultural extension workers and the language of engagement, and examine the influence of radio programmes in indigenous languages on farmers’ behaviour towards agricultural practices. The study adopted a survey research design with a questionnaire administered to 663 randomly selected farmers in Plateau, Nasarawa, and Benue states using a multistage sampling technique. Findings showed that indigenous languages play an integral role in bringing about behavioural change towards agriculture in Nigeria, with a mean Score of 3.4706 and a Standard Deviation of 1.5668. Further findings also indicate that agricultural extension workers are ready sources of information, with the language of communication being that of the local community. The study concluded that behavioural changes are evident in agricultural practices because these programmes have exposed farmers to innovations that have positively affected their agricultural practices. Thus, it is recommended that agricultural radio programmes aired in local languages should continue to be encouraged and sustained. This will help keep farmers abreast of trends in the farming sector, improving their agricultural practices.

## 1 Introduction

Indigenous languages play a crucial role in the advancement of agricultural practices. This is evident because language is a prerequisite for a human group to exist. It allows man to attain a social organisation and form. Nadal et al. [1] observe that while science has affected both linguistics and literary genres in various respects, language offers the instruments for scientific research, reporting, and dissemination. Therefore, language can only be researched as it is today with science.

Consequently, industrialised countries such as China, Japan, and Germany have almost all their technical terminologies embedded in indigenous languages. In that respect, the importance of Nigerian languages, such as Yoruba, Igbo, Hausa and many more, must be a priority to resolve illiteracy since it is necessary for profitable farming in each country to disseminate new technologies, expertise, and skills. Bamgbose [2] points out the focus on African languages in agriculture growth and development since, in his view, any attempt to do so is not only to resolve the main objective of mass participation in the sector but also to limit the benefits of international industry and investment towards a select region.

Radio programmes are essential for many developmental initiatives in developing countries, including encouraging participatory democracy and rural and community growth [3]. However, agricultural communication and the expansion of these radio stations take more airtime. This is understandable and acceptable because radio stations in rural regions of Africa have a predominantly agrarian clientele, and radio stations are always conscious that information on agriculture is most required by rural communities where local radio stations function [4].

Communication and growth specialists argue that radio is the best tool to reduce poverty and emancipate rural populations [[5], [6], [7]]. The results of research into the use of radio in agricultural communication are typical in developing nations. Most of these studies indicate that farmers are subjected to agricultural extension messages with different levels of understanding and behavioural changes. These results highlight the fundamental principles of the diffusion theory, although most such studies do not claim to have tested the distribution method [3].

The power of radio as an extension instrument lies in the comparative benefit that it can reach illiterate rural people, even in the most remote regions. Radio programmes also give people in rural areas information in the languages they know about all elements of agriculture [4]. Rural and community radio combines culture and traditional communication channels with messages about growth [8].

African communities are more likely to survive if people can communicate in their native tongues [9]. Kamalu and Atoma [10] note that because language serves as a means of communication for people, it is essential in every aspect of human endeavour. Face-to-face interactions and strategies for mass mediation are all examples of this type of communication. Considering this, employing indigenous languages to communicate with agricultural communities through the media, mainly radio, fosters a sense of community among listeners [11,12]. Studies have shown that using the native language in radio broadcasts is the most effective way to spread mass messages since it does more with less effort than other media and is understood by the audience [9,[13], [14], [15], [16], [17]]. This study thus investigates the interrelationship between indigenous languages, agricultural radio programmes and agricultural practices in Nigeria, focusing on three states (Benue, Nasarawa and Plateau). The following research objectives guide the study: to ascertain farmers' awareness of radio programmes in indigenous languages, identify farmers' sources of information, ascertain farmers' access to agricultural extension workers and the language of engagement, and examine the influence of radio programmes in indigenous languages on farmers’ behaviour towards agricultural practices.

## 2 Methodology

### 2.1 Research design

The study adopts the survey research method, a descriptive research sub-category. This research method aims to collect data on a particular problem from a sample population. The survey design allows mass communication researchers to measure the characteristics or behaviours of a sample group and then generate feedback for the population under survey [18]. It serves as the blueprint which specifies how data was collected and analysed in this study. Thus, the questionnaire and interview tools of the survey method were used to collect the necessary data for this study.

The questionnaire was designed to elicit general information about the study objectives from respondents. The choice of using the questionnaire was based on the effectiveness of the instrument o in obtaining diverse opinions and feelings from the sampled respondents. The questionnaire was delivered in person in English to the respondents with the help of research assistants who understand the language and can interpret it to farmers in their language where the need arises.

### 2.2 Population, sample size and technique

Plateau, Nasarawa, and Benue are the selected areas of the study; the states are in Nigeria's North Central geopolitical zone. It has an approximate population of 1,150,000 farmers, as indicated by the All-Farmers Association. A sample of 221 from each of the selected states was chosen randomly, giving a total of 663 farmers. The sample size was ascertained using the Krejcie and Morgan [19] sample size determination table.

The multistage sampling technique was used to get respondents. It was done through the following processes: Firstly, the geopolitical zone under focus is acknowledged as the highest contributor to Nigeria's foreign exchange earnings in agricultural crop export [20]. This zone's crops include maize, yam, cassava, soya beans, melon, rice, and tomatoes. The three top states in the geopolitical region known for successful crop farming (Benue, Nasarawa and Plateau States) were examined. Additionally, the states emphasise agricultural radio programmes in indigenous languages compared with other States in the geopolitical zone [13].

Secondly, Benue State is divided into three zones: Benue South, North-West, and Benue East. For Nasarawa State, they include Nasarawa West, Nasarawa South, and Nasarawa North. South, Central, and North Plateau make up the Plateau. Using the lottery method of simple random sampling, the researchers chose two agricultural zones from each state under investigation. The names of the farming zones were written on pieces of paper, folded appropriately, placed in a container, and vigorously shaken before being chosen. Volunteers chose the first two zones for each state.

Thirdly, each state's agricultural zones have Local Government Areas that are geographically comparable. The simple random sampling lottery method also included the random selection of one local government district. Before any picking, the names of the local governments were written on pieces of paper, folded properly, placed in a container, and thoroughly mixed. Two were chosen by a volunteer, ensuring the local administrations from each research zone were represented.

Finally, the researchers divided each state's local government into four agricultural communities by stratifying the local governments into two farming communities. These rural villages from the two senatorial zones and local governments in the State were chosen to give the researcher the needed coverage for the study. The names of the local governments were written on pieces of paper and then appropriately folded, put in a container and thoroughly mixed before any picking. A volunteer picked two, automatically comprising the local governments from each zone for the study. F.

**Fig:1:**
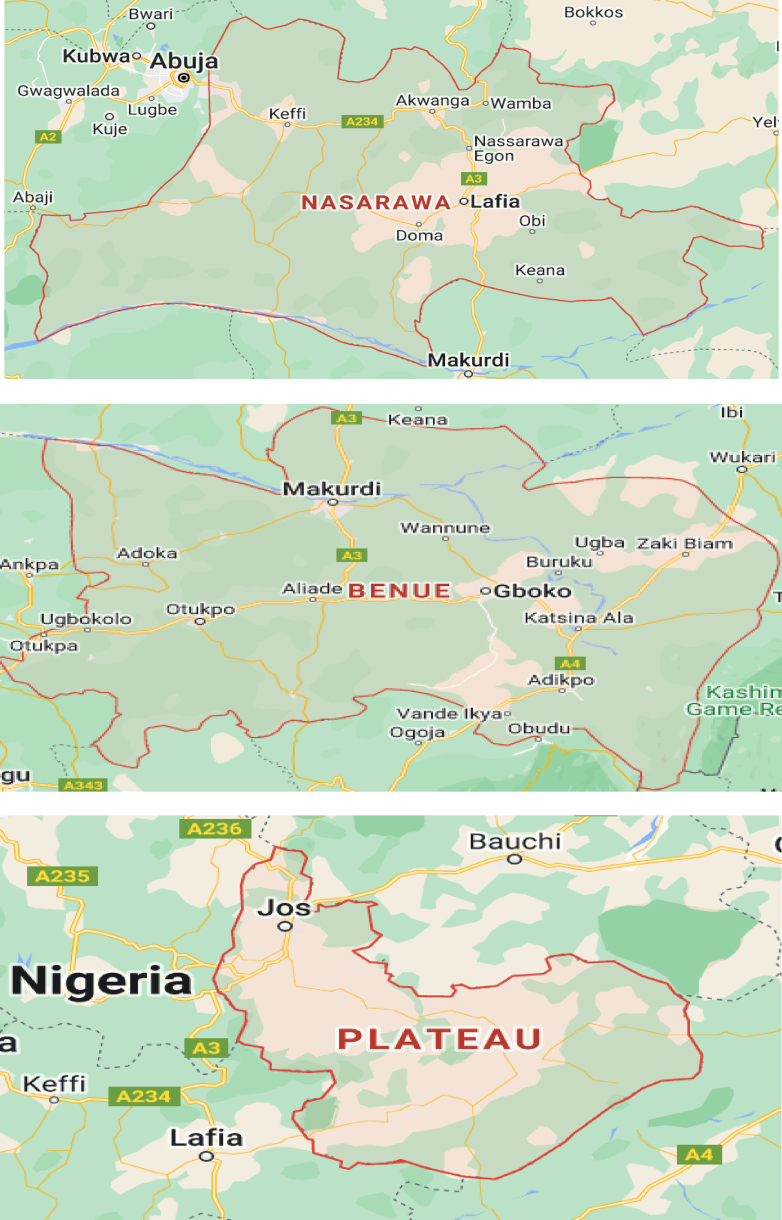
Graphical presentation of the research area.

Source: Map Data, 2024

### 2.3 Data analysis procedures

Data were analysed using tables of frequency and simple percentages to show the distribution of results and understanding of the analysis.

## 3 Ethical consideration

All data obtained during the analysis were confidential and were used exclusively for this report. NHREC N0 – IORG0010037 and CHREC Protocol Assigned Number CHREC/021/2019 were obtained from the Covenant Health Research Ethics Committee (CHREC) for ethical consent. Parents sought parental consent so their children could participate in the research. This applied to children below 18 years of age.

## 4 Results

Six hundred sixty-three (663) copies of the questionnaires were administered among the farmers across the three selected states who provided the data analysed in this section. The return rate for the questionnaire was 100 %, and it was made up of 663 questionnaires comprising data analysed in this study.

Data from Table 1 shows that the fewest respondents fall below 18 years of age, and most of this age group are from Benue, followed by Plateau and then Nasarawa. It can thus be implied that more farmers in Benue State are introduced to farming at a very young age, which is against what is compared with Plateau and Nasarawa States. It can also be inferred that respondents within this age grade are less than others, probably because those in this category have yet to decide to go entirely into farming or face other areas of human endeavour. They may not be mature enough to make informed decisions on what to do with their lives since they are still young.

**Table 1:** Demographic variables of Respondents.

Responses	Benue (%)	Nasarawa (%)	Plateau (%)
**Age**			
Below 18	11.3	7.7	8.1
19–35 Years	48.9	52.9	36.1
36–50 Years	29.9	28.5	34.8
51 and Above	9.9	10.9	21.0
**Gender**			
Male	58.8	58.8	46.1
Female	41.2	41.2	53.9
**Total**	**100**	**100**	**100**
**n=663**	**221**	**221**	**221**

Regarding respondents from the 19–35 years category, nearly half of the population across the three states falls within this category. However, respondents from Nasarawa State have the highest distribution (52.9 %) for this category of respondents, followed by Benue State (48.9 %) and then Plateau State (36.1 %). It can be inferred that these three states' active population is deep in farming. This is probably why Nigeria's three states are categorised as the hub of crop farming. Agriculture requires much energy, and the youths in these states are actively involved. Moreover, comprehending the content of programmes broadcast in indigenous languages should not be a challenge since this age grade can make informed decisions by accepting innovations that will increase their agricultural yield.

Additionally, many respondents fall between the ages of 36–50 years. From this category of respondents, the highest distribution is from Plateau State (34.8 %), closely followed by Benue State (29.9 %) and then Nasarawa State (28.5 %). This age grade is also considered economically viable and closely follows those between 18 and 35 years. The implication is that the active population in the three states, as shown from the data gathered, are predominantly farmers. At this stage, they have decided what to do with their future. Thus, farming is a viable option for this category of people. Hence, agricultural radio programmes in indigenous languages aired to this category of people will provide them with various farming options. Since they are more mature, they can adopt the innovations suggested to improve their productivity.

The number of respondents that fall within the age range of 51 and above has the highest distribution in Plateau State (21 %), closely followed by Nasarawa State (10.9 %) and then Benue State (9.9 %). From the fieldwork, it was observed that farming is not just for the young; even those who are advanced in age can also be involved, and when individuals cannot do it themselves, they hire extra hands to help. Age is not a barrier to farming as even older people are involved in small numbers. Here, experience comes into play since this category of respondents is far more mature than others. This implies that adopting innovations aired in indigenous languages from the three states should be seamless since these respondents have the most years of farming experience.

From the many age groups, this study identified individuals between the ages of 18 and 50 who had the highest distribution and were within the area where they could support themselves financially. The majority of the study's participants fall within this category of responses. Therefore, it would be appropriate to conclude that the study's respondents are suitable for the type of farming the country wants, especially agriculture, which will employ technologies to boost Nigerians' production and promote the export of agricultural products to other nations worldwide. A critical way to reach this goal is to inform these farmers in languages they can easily relate to.

According to the gender distribution, more men than women work as farmers in the Nigerian states of Nasarawa and Benue. In Plateau State, things are different since there are more female respondents than male respondents. This suggests that farming is a profession practised by men and women in the three states under examination. There is little difference in the proportion of men and women among the sampled respondents.

Additionally, there were more female respondents from Plateau than male ones. This proves that women are not excluded from farming and are eager to give their fair share to the growth of the agricultural industry. Another explanation for Plateau State having more women farmers than Nasarawa and Benue may be related to the cultural differences regarding women's contribution to farming between the two states. On the Plateau, women are more involved in farming than males are.

Table 2 shows the level of awareness of agricultural radio programmes in the three selected states. It was discovered that out of 221 respondents for Benue, 65.6 % posited that they had heard agricultural radio programmes, and 29.9 % had not heard agricultural radio programmes. In contrast, 4.5 % were still determining if they listened to agricultural radio programmes. Meanwhile, out of the 221 total respondents from Nasarawa, 80.5 % have heard agricultural radio programmes, and 15.4 % have yet to hear agricultural radio programmes. In comparison, only 4.1 % were curious if they listened to agricultural radio programmes. Similarly, out of 221 respondents from Plateau, 57.5 % have heard about agricultural radio programmes, and 18.6 % have yet to hear about agricultural radio programmes. In contrast, 24 % were curious if they listened to agricultural radio programmes.

**Table 2:** Farmers’ awareness of agricultural radio programmes.

Location		Yes (%)	No (%)	Not Sure (%)	Total
**States**	Benue	65.6	29.9	4.5	100
	Nasarawa	80.5	15.4	4.1	100
	Plateau	57.5	18.6	24.0	100
**Total** **n= 663**		**67.9**	**21.3**	**10.9**	**100**

More farmers in Nasarawa State are aware of agricultural programmes. Those from Benue and Plateau States follow them. A closer look at the figures shows that more than half of the farmers from any of the states are aware of agricultural programmes on the radio. On the other hand, a few respondents need to be made aware of these programmes, with the most significant coming from Benue State. It is necessary to point out that the total number of farmers unaware of these programmes is less than one-third of the total respondents. Altogether, about two-thirds of the respondents are aware of agricultural radio programmes.

Further to the descriptive statistics presented in Table 3, out of 221 respondents from Benue, 63.8 % posited that they heard about agricultural radio programmes in the indigenous language, and 30.3 % noted that they had not heard about agricultural radio programmes in the indigenous language. In contrast, 5.9 % were curious if they had listened to agricultural radio programmes in the indigenous language. Also, out of 221 respondents from Nasarawa, 60.2 % noted that they had heard about agricultural radio programmes in the indigenous language, and 29.9 % believed that they had not heard about agricultural radio programmes in the indigenous language. In contrast, 10 % were curious if they had listened to agricultural radio programmes in the indigenous language. Similarly, out of 221 respondents from Plateau, 49.3 % posited that they had heard about agricultural radio programmes in the indigenous language, and 24.4 % noted that they had not heard about agricultural radio programmes in the indigenous language. In contrast, 26.2 % were curious if they had listened to agricultural radio programmes in the indigenous language.

**Table 3:** Awareness of agricultural radio programmes in the indigenous languages.

Location		Yes (%)	No (%)	Not Sure (%)	Total
States	Benue	63.8	30.3	5.9	100
	Nasarawa	60.2	29.9	10.0	100
	Plateau	49.3	24.4	26.2	100
**Total** **n= 663**		**57.8**	**28.2**	**14.0**	**100**

More farmers in Benue State are aware of agricultural programmes that are aired in indigenous languages. Those from Nasarawa and Plateau follow them. A closer look at the figures shows that more than half of the farmers from all the states know agricultural programmes aired in indigenous languages on the radio. On the other hand, some respondents need to be made aware of these programmes, with the most significant coming from Nasarawa State. The total number of farmers unaware of these programmes must be less than one-third of the respondents. Altogether, more than half of the respondents know agricultural radio programmes aired in indigenous languages.

To know if the respondents get information on agricultural programmes from the radio, Table 4 shows that from 221 Benue respondents, 90 % affirmed that they get information on agriculture from radio programmes, and 8.2 % opined that they do not get information. In contrast, 1.8 % were curious if they got information on agriculture from the radio. Equally important to note is that out of 221 respondents from Nasarawa, 85.9 % affirmed that they get information on agriculture from radio programmes, 10.5 % opined that they do not, and 3.6 % were still determining if they get information on agriculture from the radio. Similarly, descriptive statistics also show that out of 221 respondents from Plateau, 54.8 % affirmed that they get information on agriculture from radio programmes and 21.2 % opined that they do not get information. In contrast, 24 % were still determining if they got information on agriculture from the radio.

**Table 4:** Farmers’ access to agricultural information through the radio.

Location		Yes (%)	No (%)	Not Sure (%)	Total
**States**	Benue	90.0	8.2	1.8	100
	Nasarawa	85.9	10.5	3.6	100
	Plateau	54.8	21.2	24.0	100
**Total** **n= 663**		**76.9**	**13.3**	**9.8**	**100**

More farmers in Benue State have access to agricultural information through radio. Those from Nasarawa and Plateau follow them. Results further show that over three-quarters of the farmers from all the states can access agricultural information through radio. However, a few respondents need access to agricultural information or be made aware of these programmes, the most significant number coming from Plateau State. It is necessary to the point that the total number of farmers who do not have access to agricultural information via radio is less than one-quarter of the total number of respondents.

Table 5 depicts how frequently respondents get information about agriculture from the radio. The descriptive statistics show that of the respondents from Benue, 9.5 % noted that they get information about agriculture from the radio very often, 19 % get information about agriculture from the radio often, and 7.7 % never obtain information about agriculture from the radio. In contrast, 63.8 % get information about agriculture from the radio occasionally. Out of the respondents from Nasarawa, 19.5 % noted that they get information about agriculture from the radio very often, 28.1 % get information about agriculture from the radio often, and 12.2 % never obtain information about agriculture from the radio. In contrast, 40.2 % get information about agriculture from the radio occasionally. The statistic also shows that of the respondents from Plateau, 13.1 % noted that they get information about agriculture from the radio very often, 14 % get information about agriculture from the radio often, and 15.4 % never obtain information about agriculture from the radio. In contrast, 57.5 % get information about agriculture from the radio occasionally.

**Table 5:** Frequency of information about agriculture from radio.

Location		Very often (%)	Often (%)	Occasionally (%)	Never (%)	Total
**States**	Benue	9.5	19	63.8	7.7	100
	Nasarawa	19.5	28.1	40.2	12.2	100
	Plateau	13.1	14	57.5	15.4	100
**Total** **n= 663**		**14**	**20.4**	**53.8**	**11.8**	**100**

Generally, of the respondents who affirmed that they have access to agricultural information through the radio in the selected States, 14 % noted that they get information about agriculture from the radio very often, 20.4 % get information about agriculture from the radio often, 11.8 % never obtain information about agriculture from the radio. In contrast, 58.3 % get information about agriculture from the radio occasionally.

More farmers in Benue State occasionally get information about agriculture from the radio. Those from Plateau and Nasarawa follow them. A closer look at the figures shows that more farmers from Nasarawa State often get information on agriculture from the radio. Benue and Plateau State follow in order.

Table 6 shows how often respondents access other media channels for agricultural information. Out of 221 respondents from Benue, 13.6 % access other media channels for agricultural information very often, 92(41.6 %) access other media channels for agricultural information often, and 81(36.7 %) occasionally access other media channels for agricultural information. In contrast, 18(8.1 %) never access other media channels for agricultural information.

**Table 6:** Farmers’ access to other media channels for agricultural information.

Location		Very often (%)	Often (%)	Occasionally (%)	Never (%)	Total
**States**	Benue	13.6	41.6	36.7	8.1	100
	Nasarawa	14.9	29.4	42.1	13.6	100
	Plateau	17.6	23.5	46.6	12.3	100
**Total** **n=663**		**15.4**	**31.5**	**41.6**	**11.5**	**100**

In addition, out of 221 respondents from Nasarawa, 14.9 % access other media channels for agricultural information very often, 29.4 % access other media channels for agricultural information often, and 42.1 % occasionally access other media channels for agricultural information. In contrast, 13.6 % never access other media channels for agricultural information. Also, out of 221 respondents from Plateau, 17.6 % access other media channels for agricultural information very often, 23.5 % access other media channels for agricultural information often, and 46.6 % occasionally access other media channels for agricultural information. In contrast, 12.3 % never access other media channels for agricultural information.

More farmers in Benue State access other media channels for agricultural information. Those from Nasarawa and Plateau-based farmers follow them. A closer look at the figures shows that less than half of the farmers from any state often access other media channels for agricultural information. On the other hand, almost half of the respondents occasionally access other media, with the most significant number coming from Plateau State.

Table 7 sought to know if respondents contact agricultural extension workers (seasoned farmers who were chosen and employed by the government to instruct and guide nearby farmers, leveraging their standing as farmers to engage with potential clientele) for agricultural information, out of 221 respondents from Benue, 69.7 % believed that they do contact agricultural extension workers for agricultural information, and 22.2 % noted that they do not contact agricultural extension workers for agricultural information. In contrast, 8.2 % were curious if they contacted agricultural extension workers for agricultural information. Of the 221 respondents from Nasarawa, 56.6 % believed that they contact agricultural extension workers for agricultural information, and 28.1 % noted that they do not contact agricultural extension workers for agricultural information. In contrast, 15.4 % were curious if they contact agricultural extension workers for agricultural information. It is equally important to note that out of 221 respondents from Plateau, 46.6 % believed that they do contact agricultural extension workers for agricultural information, and 19.5 % indicated that they do not contact agricultural extension workers for agricultural information. In contrast, 33.9 % were curious if they contact agricultural extension workers for agricultural information.

**Table 7:** Farmers reported contact with agricultural extension workers for agricultural information.

Location		Yes (%)	No (%)	Not Sure (%)	Total
**States**	Benue	69.7	22.2	8.2	100
	Nasarawa	56.6	28.1	15.4	100
	Plateau	46.6	19.5	33.9	100
**Total** **n=663**		**57.6**	**23.6**	**18.9**	**100**

More farmers in Benue State contact agricultural extension workers for agricultural information. Those from Nasarawa and Plateau follow them. A closer look at the figures shows that more than half of the state's farmers contact agricultural extension workers for agricultural information. On the other hand, a few respondents did not contact agricultural extension workers for details, with the most significant number coming from Nasarawa State. It is necessary to the point that the total number of farmers who do not contact agricultural extension workers for information is less than one-third of the total number of respondents. Altogether, just under two-thirds of the respondents contact agricultural extension workers for information.

Table 8 shows how frequently respondents get information about agriculture from agricultural extension workers. Out of 221 respondents from Benue, 14.5 % believed they get information about agriculture from agricultural extension workers very often, 12.2 % noted that they get information about agriculture from agricultural extension workers often, and 60.1 % occasionally get information about agriculture from agricultural extension workers. In contrast, 13.1 % noted that they need to obtain information about agriculture from agricultural extension workers. For the respondents from Nasarawa State, 13.1 % affirmed that they get information about agriculture from agricultural extension workers very often, 17.6 % noted that they get information about agriculture from agricultural extension workers often, and 41.6 % occasionally get information about agriculture from agricultural extension workers. In comparison, 27.6 % noted that they never obtain information about agriculture from agricultural extension workers. Furthermore, the results from Plateau State show that 9.5 % believed that they get information about agriculture from agricultural extension workers very often, 16.3 % noted that they get information about agriculture from agricultural extension workers often, and 55.2 % occasionally get information about agriculture from agricultural extension workers. In comparison, 0.19 % noted that they never obtained information about agriculture from agricultural extension workers.

**Table 8:** Frequency of farmers’ access to information about agriculture from agricultural extension workers.

Location		Very often (%)	Often (%)	Occasionally (%)	Never (%)	Total
**States**	Benue	14.5	12.2	60.1	13.1	100
	Nasarawa	13.1	17.6	41.6	27.6	100
	Plateau	9.5	16.3	55.2	19	100
**Total** **n=663**		**12.4**	**15.4**	**52.3**	**19.9**	**100**

More farmers in Nasarawa State often access information from agricultural extension workers. Those from Benue and Plateau-based farmers follow them. A closer look at the figures shows that less than half of the farmers from any state often access information from agricultural extension workers. On the other hand, less than half of the respondents need access to information from agricultural extension workers, with the most significant number coming from Nasarawa State. Furthermore, many farmers in Benue State occasionally access information from agricultural extension workers. They are followed by those from Plateau and Nasarawa-based farmers.

Table 9 reveals the respondents’ opinions on the readiness and availability of agricultural extension workers to disseminate agricultural information. Out of 221 respondents from Benue, 46.2 % believed that agricultural extension workers were readily available to disseminate agricultural information, and 22.6 % believed that agricultural extension workers were not available to disseminate agricultural information. In contrast, 31.3 % were unsure about the readiness and availability of the agricultural extension workers to disseminate agricultural information. In a related development, out of 221 respondents from Nasarawa, 52 % believed that agricultural extension workers were readily available to disseminate agricultural information, and 21.3 % believed that agricultural extension workers were not available to disseminate agricultural information. In contrast, 26.7 % were not sure about the readiness and availability of the agricultural extension workers to disseminate agricultural information. Besides, out of 221 respondents from Plateau, 40.3 % believed that agricultural extension workers were readily available to disseminate agricultural information, and 17.2 % believed that agricultural extension workers were not readily available to disseminate agricultural information. In contrast, 42.6 % were unsure about the readiness and availability of the agricultural extension workers to disseminate agricultural information.

**Table 9:** Availability of agricultural extension workers to farmers for the dissemination of agricultural information.

Location		Yes (%)	No (%)	Not Sure (%)	Total
**States**	Benue	46.2	22.6	31.3	100
	Nasarawa	52.0	21.3	26.7	100
	Plateau	40.3	17.2	42.6	100
**Total** **n=663**		**46.2**	**20.4**	**33.5**	**100**

As shown in Table 10, more farmers in Benue State aver to the availability of agricultural extension workers to farmers for disseminating agricultural information. They are followed by those from Plateau and Nasarawa-based farmers. A closer look at the figures shows that more than one-third of the farmers from any state agree with the availability of agricultural extension workers to farmers for disseminating agricultural information. On the other hand, about one-third of the respondents need to learn about the availability of agricultural extension workers to farmers to distribute agricultural information, with the most significant coming from Benue State.

**Table 10:** Language used by farmers to communicate with agricultural extension workers.

Location	English (%)	Indigenous Language (%)	Both (%)	Total	
States	Benue	29.0	5.4	65.6	100
	Nasarawa	45.7	17.1	37.2	100
	Plateau	18.1	31.2	50.7	100
**Total** **n=663**		**30.9**	**17.9**	**51.2**	**100**

It was in the interest of the researchers to know the language used by the respondents to communicate with agricultural extension workers. Of Benue respondents, 29 % use the English Language, 5.4 % use the indigenous language, and 65.6 % use the combination of English and Indigenous languages to communicate with agricultural extension workers. Of the respondents in Nasarawa, 45.7 % use the English language, 17.1 % use the indigenous language, and 37.2 % use the combination of English and Indigenous languages to communicate with agricultural extension workers. Of Plateau respondents, 18.1 % use the English Language, 31.2 % use the indigenous language, and 50.7 % use the combination of English and Indigenous languages to communicate with agricultural extension workers.

More farmers in Nasarawa State confirm that the language farmers use to communicate with agricultural extension workers is English. Those from Benue and Plateau-based farmers follow them. More farmers in Plateau State confirm that the language farmers use to communicate with agricultural extension workers is indigenous. Those from Nasarawa and Benue-based farmers follow them. Additionally, more farmers in Benue State confirm that the language farmers use to communicate with agricultural extension workers is English and indigenous. They are followed by those from Plateau and Nasarawa-based farmers. A closer look at the figures shows that a combination of indigenous languages and English is used more across the three states between agricultural extension workers and farmers.

The behavioural change of farmers through agricultural radio programmes in indigenous languages was measured with seven (7) items on the research instrument using five (5) Likert's scale ranging from strongly agree (5), Agree (4), undecided (3), disagree (2) and strongly disagree (1). The scale addressed the extent to which respondents perceived the items on the research instrument.

The extent to which the respondents agreed to the items on the research instrument was determined by the mean score and standard deviation. The assumptions and threshold of the mean score and standard deviation depict that if the mean is between 0.1 and 1.30, it is an indication that it is feeble; 1.31–2.40 means score shows that it is very fair, 2.41–3.45 is regarded as moderate; 3.46–4.50 is strong while 4.51–5.00 is an indication that the means score is powerful.

Table 11 depicts respondents’ opinions on the behavioural change of farmers because of agricultural radio programmes in indigenous languages. The descriptive statistic shows that 9.6 % of the respondents strongly agreed that they engage in the practices recommended by the agricultural radio programmes in indigenous languages on the local radio station, 47.7 % of the respondents agreed that they participate in the exercises recommended by the agricultural radio programmes in indigenous languages on the local radio station, 22.9 % of the respondents were indifferent about the statement, 14.9 % disagreed with the statement. In comparison, 5 % strongly disagreed with the statement (Mean Score = 3.4706, Standard Deviation = 1.5668). Therefore, most respondents agreed that they engage in the practices recommended by the agricultural radio programmes in indigenous languages on the local radio station.

**Table 11:** Influence of agricultural radio programmes in indigenous languages on farmers’ behaviour towards agricultural practices.

		SA [5] (%)	A [4] (%)	U [3] (%)	D [2] (%)	SD [1] (%)	Mean	SD
**I engage in the practices recommended by the agricultural radio programmes in indigenous languages on our local radio station.**	Benue	4.1	53.4	28.5	9.5	4.5		
	Nasarawa	14.5	48.0	14.9	16.3	6.3		
	Plateau	10.0	41.6	25.3	19.0	4.1		
	**Total**	**9.6**	**47.5**	**22.9**	**14.9**	**5.1**	**3.4706**	**1.56683**
**Agricultural radio programmes in indigenous languages on our local radio station help farmers improve their production.**	Benue	6.8	75.1	11.8	3.2	3.2		
	Nasarawa	29.0	49.8	10.4	6.8	4.1		
	Plateau	18.1	48.4	16.3	14.0	3.2		
	**Total**	**17.9**	**57.8**	**12.8**	**8.0**	**3.5**	**3.7873**	**0.94646**
**Agricultural radio programmes in indigenous languages on our local radio station help farmers embrace innovations.**	Benue	8.6	73.3	12.7	3.6	1.8		
	Nasarawa	24.9	44.3	15.8	7.7	7.2		
	Plateau	21.3	44.3	17.6	14.0	2.7		
	**Total**	**18.3**	**54**	**15.4**	**8.4**	**3.9**	**3.7421**	**0.98086**
**Agricultural radio programmes in indigenous languages on our local radio station encourage farmers to use technology in farming.**	Benue	10.0	63.8	14.5	9.0	2.7		
	Nasarawa	20.9	50.2	13.1	10.4	5.4		
	Plateau	17.2	50.7	13.6	14.5	4.1		
	**Total**	**16**	**54.9**	**13.7**	**11.3**	**4.1**	**3.7330**	**1.86061**
**Agricultural radio programmes in indigenous languages on our local radio station expose farmers to new markets for their produce.**	Benue	10.0	43.4	32.6	13.1	0.9		
	Nasarawa	19.0	50.2	11.8	14.5	4.5		
	Plateau	19.9	46.2	15.8	14.0	4.1		
	**Total**	**16.3**	**46.6**	**20.1**	**13.9**	**3.2**	**3.5897**	**1.01827**
**Agricultural radio programmes in indigenous languages on our local radio station improve farmers' general knowledge of agriculture.**	Benue	10.0	69.2	14.5	5.0	1.4		
	Nasarawa	21.7	54.8	8.6	10.9	4.1		
	Plateau	17.2	48.4	16.3	13.6	4.5		
	**Total**	**16.3**	**57.5**	**13.1**	**9.8**	**3.3**	**3.8115**	**2.20869**
**Farmers are exposed to agricultural funding through agricultural radio programmes in indigenous languages on our local radio station.**	Benue	8.1	43.0	38.0	8.6	2.3		
	Nasarawa	22.6	45.7	11.8	13.6	6.3		
	Plateau	15.4	48.4	19.5	13.1	3.6		
	**Total**	**15.4**	**45.7**	**23.1**	**11.8**	**4.1**	**3.5656**	**1.01715**

The statistics presented in the table also show that 17.9 % of the respondents strongly agreed that agricultural radio programmes in indigenous languages on the local radio station help farmers improve their production, 57.8 % of the respondents agreed that agricultural radio programmes in indigenous languages on the local radio station help farmers improve their production, 12.8 % of the respondents were indifferent about the statement, 8 % disagreed with the statement. In comparison, 23(3.5 %) strongly disagreed with (Mean Score = 3.7873, Standard Deviation = 0.9465). Therefore, most respondents agreed that agricultural radio programmes in indigenous languages on the local radio station help farmers improve their production on the local radio station.

It was also discovered that 16.3 % of the respondents strongly agreed that agricultural radio programmes in indigenous languages on the local radio station help farmers embrace innovations, 46.6 % of the respondents agreed that agricultural radio programmes in indigenous languages on the local radio station help farmers embrace changes, 20.1 % of the respondents were indifferent about the statement, 13.9 % disagreed with the statement. In comparison, 3.2 % strongly disagreed with (Mean Score = 3.7421, Standard Deviation = 0.9809). Therefore, most respondents agreed that agricultural radio programmes in indigenous languages on the local radio station help farmers embrace innovations.

Meanwhile, 16 % of the respondents strongly agreed that agricultural radio programmes in indigenous languages on the local radio station encourage farmers to use technology in farming, 54.9 % of the respondents agreed that agricultural radio programmes in indigenous languages on the local radio station encourage farmers to use technology in agriculture, 13.7 % of the respondents were indifferent about the statement, 11.3 % disagreed with the statement. In comparison, 4.1 % strongly disagreed with (Mean Score = 3.7330, Standard Deviation = 1.8606). Therefore, most respondents agreed that agricultural radio programmes in indigenous languages on the local radio station encourage farmers to use technology in farming.

Also, 16.3 % of the respondents strongly agreed that agricultural radio programmes in indigenous languages on the local radio station expose farmers to new markets for their produce, 46.6 % of the respondents agreed that agricultural radio programmes in indigenous languages on the local radio station expose farmers to new markets for their food, 20.1 % of the respondents were indifferent about the statement, 13.9 % disagreed with the statement. In comparison, 3.2 % strongly disagreed with (Mean Score = 3.5897, Standard Deviation = 1.0183). Therefore, most respondents agreed that agricultural radio programmes in indigenous languages on the local radio station expose farmers to new markets for their produce. Similarly, 16.3 % of the respondents strongly agreed that agricultural radio programmes in indigenous languages on the local radio station improve farmers' general knowledge of agriculture, 57.5 % of the respondents agreed that agricultural radio programmes in indigenous languages on the local radio station improve farmers general knowledge of agriculture, 13.1 % of the respondents were indifferent about the statement, 9.8 % disagreed with the statement. In comparison, 3.3 % strongly disagreed with (Mean Score = 3.8115, Standard Deviation = 1.20869). Therefore, most respondents agreed that agricultural radio programmes in indigenous languages on the local radio station improve farmers’ general knowledge of agriculture.

In a related development, 15.4 % of the respondents strongly agreed that farmers are exposed to agricultural funding through agricultural radio programmes in indigenous languages on the local radio station, 45.7 % of the respondents agreed that farmers are exposed to agricultural financing through agricultural radio programmes in indigenous languages on the local radio station, 23.1 % of the respondents were indifferent about the statement, 11.8 % disagreed with the statement. In comparison, 4.1 % strongly disagreed with (Mean Score = 3.5656, Standard Deviation = 1.0172).

It must also be noted that the respondents’ opinions on the behavioural change of farmers through agricultural radio programmes in indigenous languages across the selected states are similar (see Table 11). Therefore, most respondents agreed that farmers are exposed to agricultural funding through agricultural radio programmes in indigenous languages on the local radio station.

## 5 Discussion

Findings relating to the level of awareness of agricultural radio programmes in the three selected states show that more farmers in Nasarawa State are aware of agricultural programmes than those from the other states. More than half of the farmers from any state are aware of agricultural programmes on the radio. On the other hand, a few respondents need to be made aware of these programmes, with the most significant coming from Benue State. The findings of Nyamekye et al. [21], Ado et al. [22], and Bentley et al. [23] all allude to the fact that awareness of programmes on radio adds value to the desire to get the required change and that agricultural programmes in local languages hosted online were crucial for sustainable development in agriculture. They continued by saying that all these farmer learning programmes provide helpful information on sustainable agricultural innovations to inspire farmer experiments and that farmers are now beginning to use the internet independently to conduct proactive information searches.

More farmers in Benue State have access to agricultural information via radio. Results also indicate that radio is a source of agricultural knowledge for more than 75 % of farmers across all states. However, only a small percentage of respondents, the majority from Plateau state, need access to agricultural knowledge or are unaware of these programs. It is necessary to the extent that less than 25 % of the respondents overall are farmers who need access to agricultural information via radio. This is supported by the findings of Khan et al. [24] and Adio et al. [25]) who demonstrated that access to agricultural information is critical to agricultural development. Findings relating to the frequency of information on agriculture provided by the radio indicate that more farmers in Benue receive this information on a sporadic basis. This aligns with the findings of Popoola et al. [26], who revealed that the frequency of listenership to radio programmes was a source of agricultural information, which provided the needed information for agricultural development, thus bringing about a behavioural change towards farming. Furthermore, Adamides and Stylianou [27] affirmed that farmers were perceived to align with the effectiveness of the farm broadcast category in the transfer of agricultural technology. The majority were satisfied with the farm broadcasts' timing and wanted the duration to be increased to 1 h. They suggested using the local language during the broadcasting of the programme and live broadcasts of discussions with agriculture scientists and successful entrepreneurs.

Findings indicate that more farmers in Benue State consult other media outlets for agricultural information than farmers in other states. Farmers from the Plateau and Nasarawa follow them. A detailed examination of the data reveals that less than half of the farmers in each state frequently visit other media sources for agricultural information. On the other hand, more than half of the respondents reported not using any other media, with Plateau State having the highest percentage. This finding truly reflects what is obtainable from farmers and other information sources as they rely on something other than what they listen to on the radio. This is exemplified in the findings of Adio et al. [25], which show that information sources and services used mainly by the farmers included relations, fellow farmers, town criers, television, mobile phones, the film shows, and radio, among others. Thus, most farmers rely on informal sources of information from neighbours, friends, and colleagues rather than from agricultural extension workers.

Extension agents are essential sources of agricultural data. To obtain agricultural information, the survey discovered that more farmers in Benue State contact extension agents. Additionally, research reveals that more than half of the state's farmers contact extension agents for advice on farming. Most responders from Nasarawa State do not contact agricultural extension workers for information. It is essential to the extent that fewer than one-third of farmers who responded to the survey do not seek information from extension agents. About two-thirds of all respondents contact agricultural extension workers for information. To elaborate further, research shows that Nasarawa State farmers access extension services more frequently. Farmers from the Plateau and Benue follow them. Less than half of the farmers in each state regularly utilize agricultural extension workers' information. Less than half of the respondents, mainly from Nasarawa State, do not use agricultural extension workers to obtain information. Additionally, more farmers in Benue State periodically consult extension specialists for information. This is supported by Aphunu and Otoikhian [28] and Olorunfemi et al. [29]. They argued that farmers perceived extension agents to be vast in knowledge of the subject matter and that they integrated theories with practical knowledge well.

Findings about farmers’ views on agricultural extension workers' preparedness and availability to communicate agricultural information show that agricultural extension workers are readily available to them for disseminating agricultural information in Benue State. More than one-third of farmers from each state mention the availability of agricultural extension workers to distribute agricultural information to farmers. On the other hand, a small percentage of respondents, the majority of whom are from Benue state, are unaware that agricultural extension workers are available to farmers to deliver agricultural knowledge. This result conflicts with Aphunu and Otoikhian [28], who noted that farmers were unimpressed with extension agents' abilities to teach and communicate. The adoption of technologies and the efficiency of extension agents were significantly correlated. Thus, it could be extrapolated that it was one of the factors that led farmers to choose to use other resources for agricultural knowledge. The results, however, corroborate Msuya et al. [30] that providing the farming community with knowledge on new technology they might adopt to raise productivity, incomes, and standards of life plays a crucial role in African development. Furthermore, Olorunfemi et al. [31] concur that extension agents participate in the dissemination of climate-smart agricultural initiatives, such as water management initiatives, tillage innovative initiatives, fossil fuel burning reduction initiatives, soil initiatives, crop-mix initiatives, and ICT/other technological initiatives to help farmers increase productivity.

According to the study's findings regarding the language of communication, farmers in Nasarawa State concur that they speak English while speaking with extension personnel. More farmers in Plateau State attest that they converse with extension agents in their native tongue. A growing number of farmers in Benue State also attest that they communicate with extension agents in English and local languages. Farmers from the Plateau and Nasarawa come after them. As a result, extension agents and farmers in the three states speak a blend of indigenous languages and English more frequently. Moyo and Salawu [32] corroborate this finding. They underscored the importance of speaking to farmers in a language they understand for improved productivity and technological transfer.

Findings relating to the behavioural change of farmers through agricultural radio programmes in indigenous languages show that most respondents agreed that they engage in the practices recommended by the agricultural radio programmes in indigenous languages on the local radio station. Furthermore, 75.7 % of respondents agree that agricultural radio programmes are in indigenous languages. It was also discovered that most respondents agreed that agricultural radio programmes in indigenous languages on the local radio station help farmers embrace innovations. This finding is corroborated by Tambo et al. [33], who found that the media are veritable tools for enhancing behavioural change among farmers.

Meanwhile, 70.9 % of the respondents affirm that agricultural radio programmes in indigenous languages on the local radio station encourage farmers to use technology in farming. Also, findings show that 62.9 % of the respondents affirm that agricultural radio programmes in indigenous languages on the local radio station expose farmers to new markets for their produce. Similarly, most respondents agree that agricultural radio programmes in indigenous languages on the local radio station improve farmers’ general knowledge of agriculture. In a related development, most respondents agreed that farmers are exposed to agricultural funding through agricultural radio programmes in indigenous languages on the local radio station. These findings are supported by Salik et al. [34]. Their study found a correlation between the dissemination of information through radio programmes and increased agricultural production.

Agwu et al. [35] support that farm broadcasting was farmers' primary information source. They also noted significant limitations, including brief programme length, inappropriate programme scheduling, failure to ask appropriate questions and feedback from the radio presenter and language used to present the programme. The study holds sway in this research work because it emphasises that farm broadcasts were the significant sources of information for a higher proportion of the farmers. The language of the presentation of agricultural radio programmes was identified as a significant constraint. This constraint is a departure from this study's findings, which bring indigenous languages to the fore in agricultural radio programmes.

The findings of Muhammed and Olabode [36], who examined the efficiency of two long-running agricultural programmes, show a favourable evaluation of both programme elements and the importance of the two programmes in improving agricultural output across 12 perceptual dimensions. Even though most farmers presently use radio more than television, they recognise that TV programmes provide helpful content. Haider's [37] finding that local agricultural radio programmes influence their buying behaviour further supports this study. The research results show that farmers are adopting new farming methods through local radio information. The study also shows that local radio programmes help farmers embrace news and apply new techniques and procedures on their farms. Evidence shows that if more farmers listen to agricultural radio programmes, they will welcome innovations in their farms, and expansion will take place in their farms.

## 6 Conclusion and recommendations

The findings in this study portray the potential of indigenous languages in agricultural radio programmes and behavioural change towards agricultural practices in Nigeria. The study concludes that farmers know agricultural programmes aired in indigenous languages. The study underscores improved agricultural practices through agricultural radio programmes in indigenous languages. This is evident in behavioural changes because these programmes have exposed farmers to innovations affecting their agricultural practices.

Based on the findings of the study and the conclusions reached, the following recommendations are made:

a. Agricultural radio programmes aired in local languages should continue to be encouraged and sustained. This will help keep farmers abreast of trends in the farming sector, improving their agriculture practices.
b. Farmers should also contact suitable sources for agricultural information. This will go a long way to foster agricultural productivity. The government could develop a call centre where farmers can quickly call to make enquiries that will help them. Conversations with the call centre should be in indigenous languages for easy understanding
c. Access to agricultural extension workers should also be maintained. They should be readily available by providing mobility so they can seamlessly attend to the needs of farmers.
d. Other minority languages spoken in hinter communities should be given media coverage in agricultural radio programmes.

## Data availability

Data will be made available on request.

## CRediT authorship contribution statement

**Babatunde Adeyeye:** Writing – review & editing, Writing – original draft, Conceptualization. **Abiodun Salawu:** Writing – review & editing, Supervision. **Evaristus Adesina:** Methodology, Investigation.

## Declaration of competing interest

The authors declare that they have no known competing financial interests or personal relationships that could have appeared to influence the work reported in this paper.
